# Invasive ductal carcinoma of breast and squamous cell carcinoma of anterior chest wall—A rare collision

**DOI:** 10.1002/ccr3.2964

**Published:** 2020-05-22

**Authors:** Dhiraj Mallik, Bina Ravi, Navin Kumar, Debarati Chattopadhyay, Anjum Syed, Prashant Joshi

**Affiliations:** ^1^ Department of Breast Surgery AIIMS Rishikesh India; ^2^ Department of Plastic Surgery AIIMS Rishikesh India; ^3^ Department of Radiodiagnosis AIIMS Rishikesh India; ^4^ Department of Pathology AIIMS Rishikesh India

**Keywords:** breast, chest wall, collision tumors, Invasive ductal carcinoma, squamous cell carcinoma

## Abstract

Collision tumors are two distinct neoplasms seen together in same anatomic site. Management of such rare entity still lacks standardization with unknown prognosis. Here we are presenting one such rare case of invasive ductal carcinoma of breast and squamous cell carcinoma of anterior chest wall in a 31‐year‐old lady.

## Graphical abstract

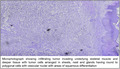

## 1 CASE DESCRIPTION

**Figure 1:**
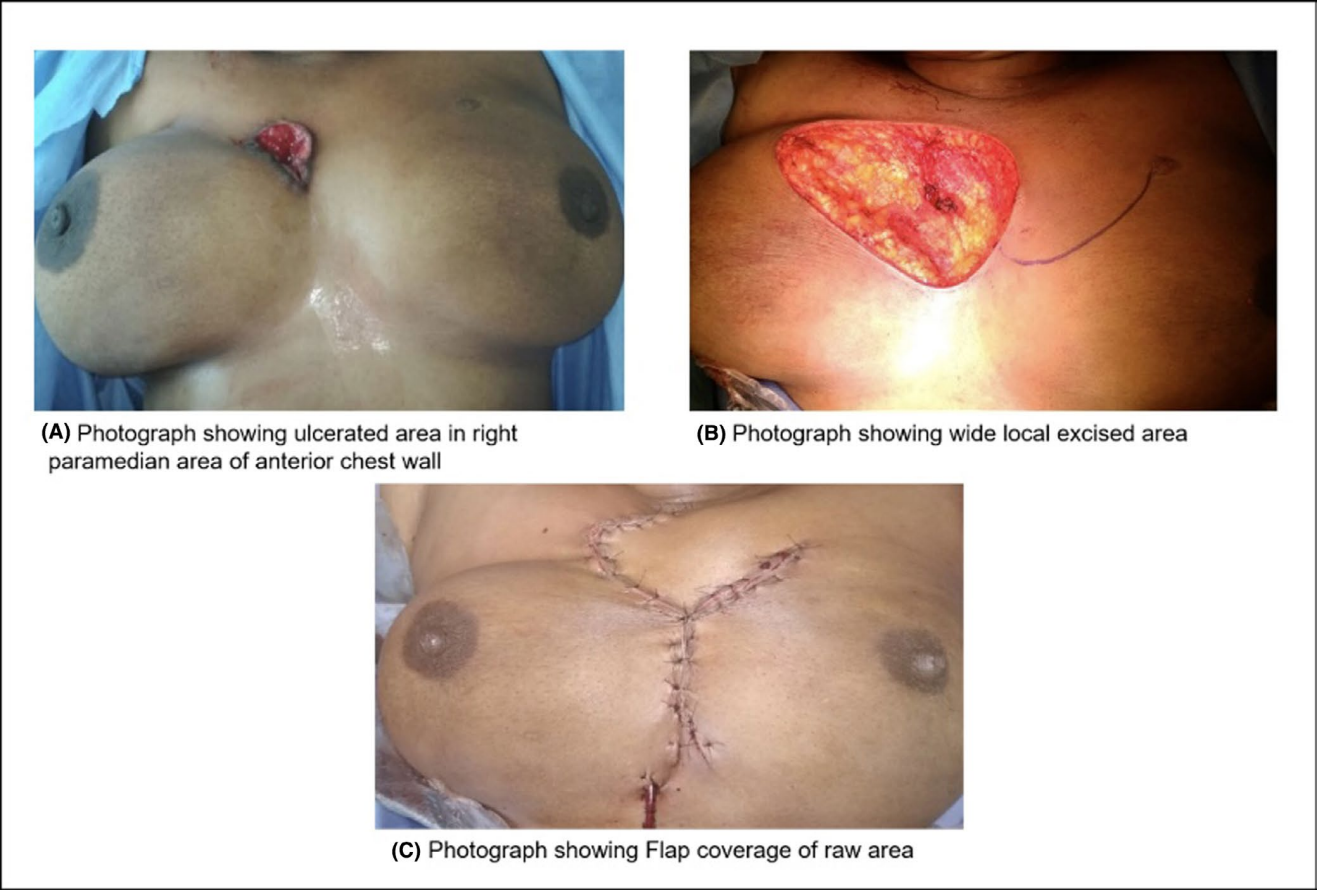
Clinical Pictures

A 31‐year‐old female came to OPD with a complaint of nodule in paramedian position of chest wall toward right breast for past 2 years. The lesion was progressive in nature which developed into an ulcer associated with pain and serous discharge (Figure 1). No history of any lump in right breast or discharge from right nipple. Family history was insignificant. On local examination, an ulcer in anterior chest wall measuring 5 × 3 × 1 cm with slough and not adherent to underlying structures. No palpable lump in bilateral breast. Patient's blood parameters were within normal limit.

Mammography revealed BIRADS 1, negative study. Contrast‐enhanced computed tomography (CECT) chest showed well‐defined lobulated nonenhancing lesion (32 × 34 mm) situated in subcutaneous plane in anterior chest wall in right paramedian location with mild skin thickening and ulceration with bilateral axillary lymphadenopathy. Contrast‐enhanced magnetic resonance imaging (CE‐MRI) breast showed a well‐defined cystic lesion with thick enhancing wall with speculated margins in subcutaneous planes of midline and right paramedian area of anterior chest wall with a thin linear tract from the lesion reaching up to the skin.

Wedge biopsy from the lesion was taken, and histopathological examination (HPE) report suggested squamous cell carcinoma.

**Figure 2:**
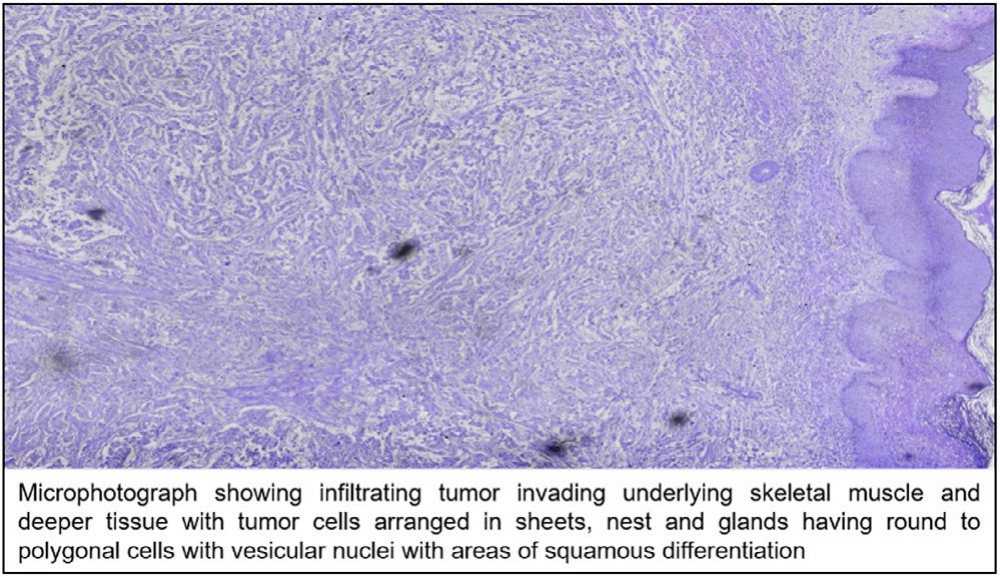
H&E (20X)

Patient then underwent wide local excision (Figure 1B) with flap reconstruction (Figure 1C) and excised specimen sent for HPE. The report suggested two differential diagnoses as of invasive ductal carcinoma with squamous differentiation grade II or squamous cell carcinoma (SCC) (Figure 2).

On immunohistochemistry panel ER, PR, Her2neu were positive, p63 focally positive in tumor cells and Ki67 25%‐30% giving diagnosis of invasive ductal carcinoma with squamous differentiation. Decision for radiation therapy for chest wall SCC followed by definitive therapy for breast carcinoma was taken, and patient was sent to radiotherapy department after which patient lost to follow up.

## 2 DISCUSSION

Collision tumors are uncommon clinicopathological entities where two different malignant tumors having distinct histology coinciding in same anatomic site.^1^ It can be seen in same organ or metastasis from other sites. These neoplasms are synchronous in nature as described by Meyer et al^2^ This is similar to our present case. As described by Spagnolo et al, there are three diagnostic parameters such as (a) two separate site of origin, (b) dual origin should be recognized, and (c) transitional patterns at the site of collision.^3^

**Figure 3:**
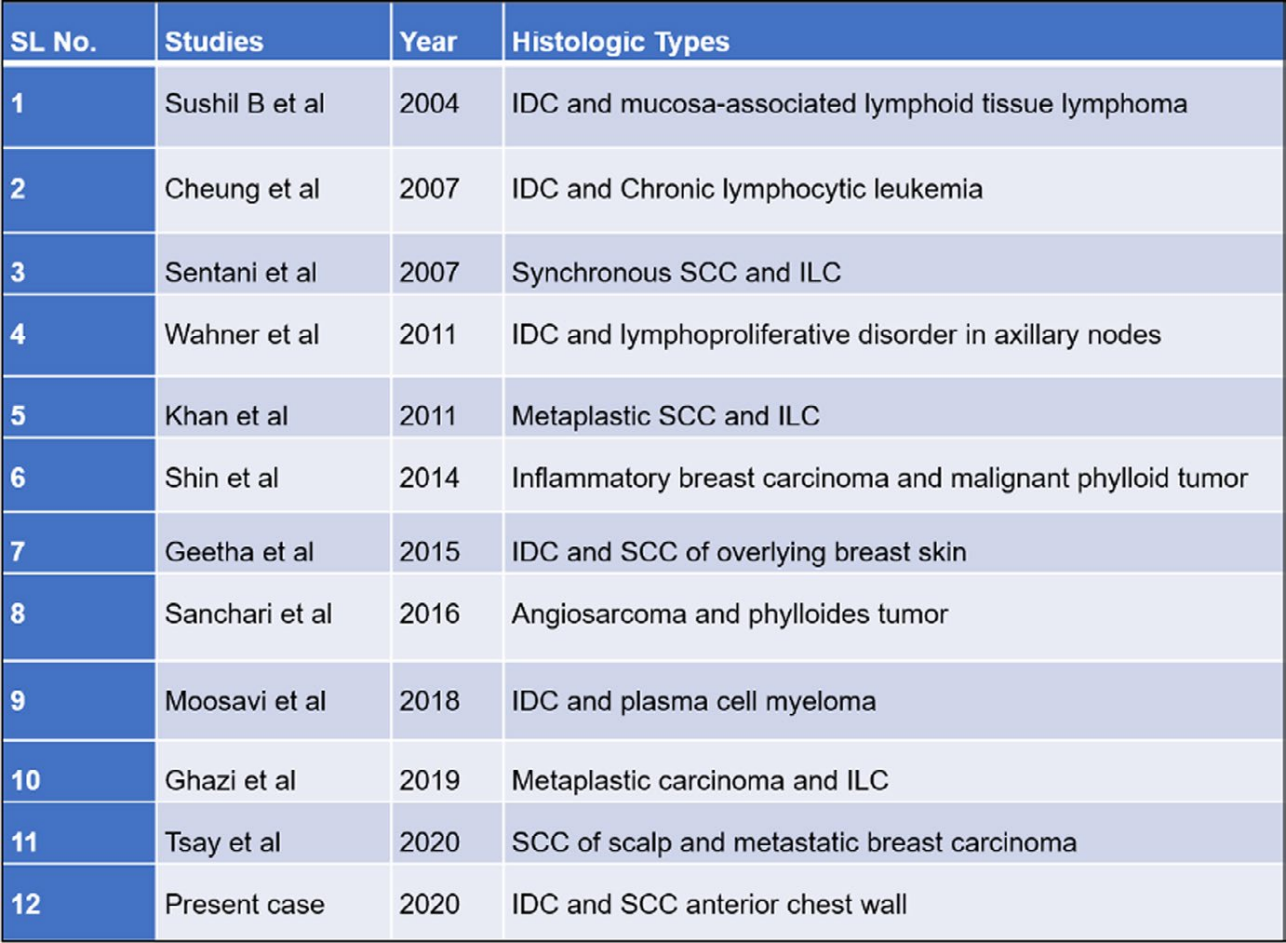
Existing case reports on collision tumors of breast

**Figure 4:**
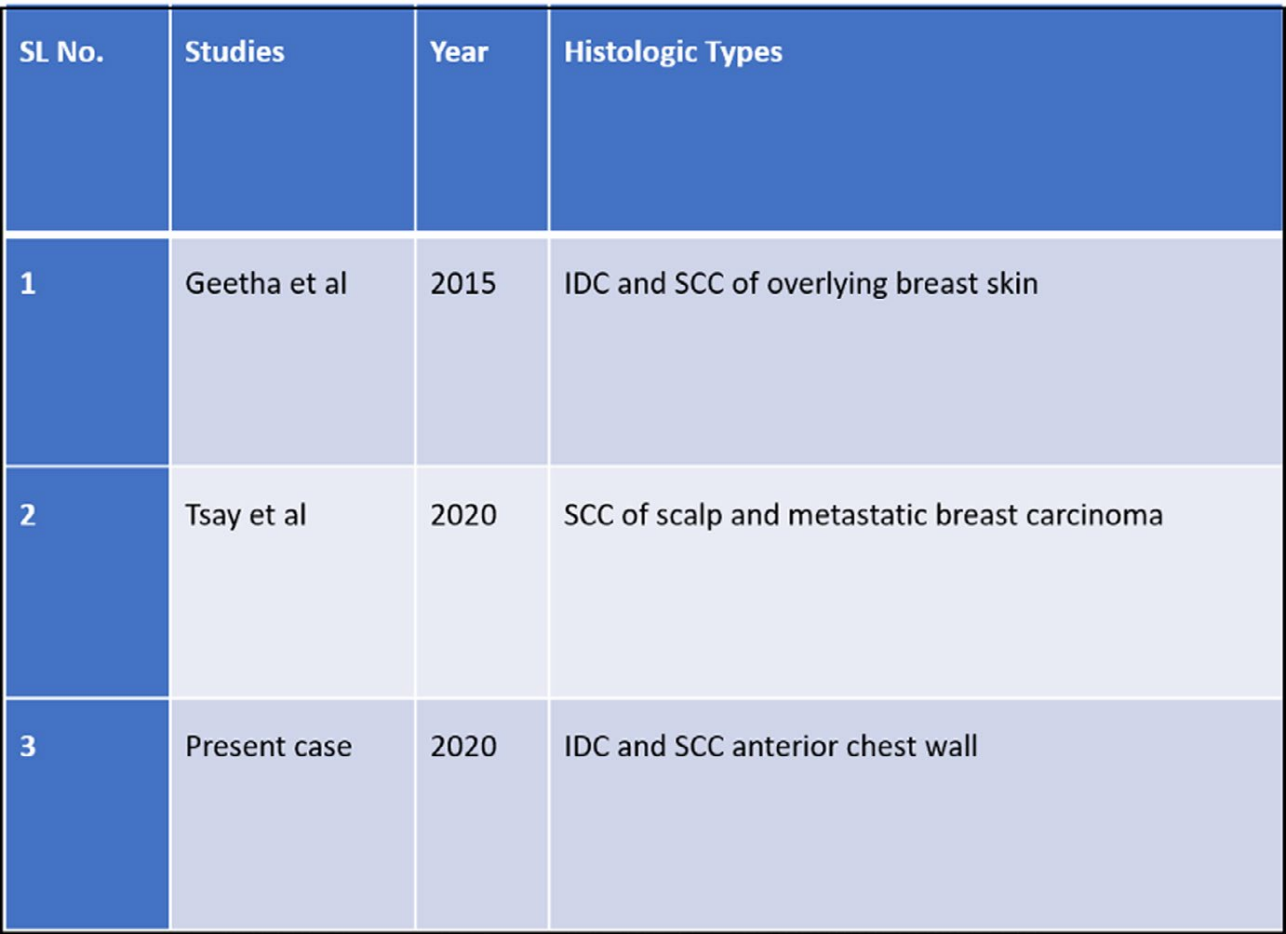
Existing case reports on breast carcinoma and cutaneous SCC

Occurrence of such entities in breast is rare. Only 11 cases have been reported in literature (Figure 3). Usually seen in 4th to 6th decade of life with female preponderance. Incidence of such entities is still a mystery.^4^ Breast carcinoma and cutaneous SCC is a very rare collision, and in literature, only 2 cases have been reported till now.^4^, ^5^ To the best of our knowledge, our case is 3rd of this kind (Figure 4). Concept of overexpression of p53 has been described by Filippakis et al as pathogenesis of collision tumor.^6^

Cutaneous SCC and primary SCC from breast is differentiated by various IHC markers one of which is p63 which is positive for cutaneous SCC.^7^ This is similar to our case. For breast carcinoma, hormonal receptor status is most crucial investigation which will lead to its prognostication. In our case, it is triple‐positive.

Management of these entities is still not standardized. In the present case, diagnosis could only be reached postoperatively on IHC. Multidisciplinary approach should always be present because sometimes we are not aware of the aggressive component. Other ancillaries such as histology and stage might help to prognosticate the entity.

## 3 CONCLUSION

On extensive search of literature, we can conclude that this case might be the third reported case. It should be always kept in mind that such rare case can occur in nature, and we should be able to manage and prognosticate the disease for the betterment of the patient by multidisciplinary approach.

## CONFLICT OF INTEREST

None declared.

## AUTHOR CONTRIBUTIONS

DM: collected data, structured the study and design, prepared the manuscript; BR: helped structuring the study and design and drafting the manuscript; NK: helped structuring the study and design and drafting the manuscript; DC: helped structuring the study and design and drafting the manuscript; AS: evaluated radiological data; PJ: evaluated pathological data.

## References

[1] Shin YD , Lee SK , Kim KS , et al, Collision tumor with inflammatory breast carcinoma and malignant phyllodes tumor: a case report and literature review. World J Surg Oncol. 2014;12:5. 24400686 10.1186/1477-7819-12-5 PMC3895737

[2] Meyer R . Beitrag zur verstandigung uber die namengebung in dergeschwulstlehre. Zentralbl Allg Pathol. 1919;30:291‐296.

[3] Spagnolo DV , Heenan PJ . Collision carcinoma at the esophagogastric junction: report of two cases. Cancer. 1980;46:2702‐2708. 7448709 10.1002/1097-0142(19801215)46:12<2702::aid-cncr2820461228>3.0.co;2-m

[4] Geetha R , Kalyani R , Srinivas MV , Shakthidasan C . A rare collision tumour of infiltrating ductal carcinoma and squamous cell carcinoma of skin overlying breast: a case report. J Clin Diagn Res. 2015;9(1):XD06–XD08. 10.7860/JCDR/2015/10437.5464 PMC4347163 25738072

[5] Tsay AJ , Paine AR , Lighthall JG , Choi KY , Hebel J , Flamm A . A possible pitfall of Mohs surgery in collision tumor diagnosis: a case of a squamous cell carcinoma of the scalp overlying a metastatic breast lesion of the skull. JAAD Case Rep. 2020;6(2):119‐121. 32016155 10.1016/j.jdcr.2019.12.004 PMC6992889

[6] Filippakis GL , Lagoudianakis EE , Genetzakis M , et al, Squamous cell carcinoma arising in a mature cystic teratoma of the ovary with synchronous invasive lobular breast cancer: case report. Eur J Gynaecol Oncol. 2006;27:537‐540. 17139997

[7] Compton LA , Murphy GF , Lian CG . Diagnostic immunohistochemistry in cutaneous neoplasia: an update. Dermatopathology. 2015;2:15‐42. 27047932 10.1159/000377698 PMC4816435

